# Transcriptional profiling and targeted proteomics reveals common molecular changes associated with cigarette smoke-induced lung emphysema development in five susceptible mouse strains

**DOI:** 10.1007/s00011-015-0820-2

**Published:** 2015-05-12

**Authors:** Maciej Cabanski, Brett Fields, Stephanie Boue, Natalia Boukharov, Hector DeLeon, Natalie Dror, Marcel Geertz, Emmanuel Guedj, Anita Iskandar, Ulrike Kogel, Celine Merg, Michael J. Peck, Carine Poussin, Walter K. Schlage, Marja Talikka, Nikolai V. Ivanov, Julia Hoeng, Manuel C. Peitsch

**Affiliations:** Philip Morris International Research and Development, Philip Morris Products S.A, Quai Jeanrenaud 5, 2000 Neuchâtel, Switzerland; Selventa, One Alewife Center, Cambridge, MA 02140 USA; Novartis Pharma AG, Novartis Institutes for Biomedical Research (NIBR), 4002 Basel, Switzerland; Bayer Technology Services GmbH, 51368 Leverkusen, Germany

**Keywords:** Emphysema, Cigarette smoke, Systems biology, Mouse models, Gene expression, Molecular mechanisms

## Abstract

**Background:**

Mouse models are useful for studying cigarette smoke (CS)-induced chronic pulmonary pathologies such as lung emphysema. To enhance translation of large-scale omics data from mechanistic studies into pathophysiological changes, we have developed computational tools based on reverse causal reasoning (RCR).

**Objective:**

In the present study we applied a systems biology approach leveraging RCR to identify molecular mechanistic explanations of pathophysiological changes associated with CS-induced lung emphysema in susceptible mice.

**Methods:**

The lung transcriptomes of five mouse models (C57BL/6, ApoE^**−/−**^, A/J, CD1, and Nrf2^**−/−**^) were analyzed following 5–7 months of CS exposure.

**Results:**

We predicted 39 molecular changes mostly related to inflammatory processes including known key emphysema drivers such as NF-κB and TLR4 signaling, and increased levels of TNF-α, CSF2, and several interleukins. More importantly, RCR predicted potential molecular mechanisms that are less well-established, including increased transcriptional activity of PU.1, STAT1, C/EBP, FOXM1, YY1, and N-COR, and reduced protein abundance of ITGB6 and CFTR. We corroborated several predictions using targeted proteomic approaches, demonstrating increased abundance of CSF2, C/EBPα, C/EBPβ, PU.1, BRCA1, and STAT1.

**Conclusion:**

These systems biology-derived candidate mechanisms common to susceptible mouse models may enhance understanding of CS-induced molecular processes underlying emphysema development in mice and their relevancy for human chronic obstructive pulmonary disease.

**Electronic supplementary material:**

The online version of this article (doi:10.1007/s00011-015-0820-2) contains supplementary material, which is available to authorized users.

## Introduction

Chronic obstructive pulmonary disease (COPD) is a complex pulmonary disorder primarily induced by cigarette smoking and characterized by poorly or not reversible airflow obstruction. The three major clinical manifestations of COPD are emphysema, chronic bronchitis and small airway diseases [1, 2]. A hallmark of the disease is chronic inflammation in the airways and lungs, which together with protease/anti-protease imbalance, apoptosis and oxidative stress contributes to the progressive tissue damage underlying COPD pathophysiology [3, 4]. Increasing severity of the disease, evidenced by more pronounced airflow obstruction, is measured by spirometry and graded according to the Global Initiative for chronic obstructive lung disease (GOLD) criteria (grades I–IV). Grades I and II represent mild and usually early stages of COPD, while grades III and IV correspond to progressively more severe disease stages [2].

Our understanding of emphysema and COPD pathogenesis has evolved considerably over the past 50 years; however, we still have very limited knowledge regarding the complex molecular mechanisms that drive the pathological changes. Although >90 % of COPD patients are smokers, only 20 % of smokers develop COPD [5, 6] with the underlying reasons for this being complex but likely involving genomic variation as an important determinant of disease susceptibility [7]. In mice, CS-related pulmonary changes also have a genetic component, evidenced by the fact that different strains display varying susceptibility to CS exposure and emphysema development [8–10]. It has been proposed that differential susceptibility or resistance of these strains to CS exposure may be due to the varying degree of activation of signaling pathways such as nuclear factor (NF)-κB, p38 mitogen-activated protein kinase or histone deacetylase (HDAC)2 [8–10]. In addition, genetic or pharmacological manipulation resulted in identification of many key molecules associated with murine CS-induced emphysema [11]. Several groups, using knock-out and transgenic mice, have clearly demonstrated the importance of multiple genes and pathways, including chemokine/cytokine receptors (e.g. C–C motif receptor (CCR)1, CCR5, CCR6 [12–14]), metalloproteinase enzymes (e.g. MMP-9/12 [15]), cytokines (e.g. interleukin (IL)-1β, IL-18 [16, 17]), and transcription factors (TFs) such as NF-κB [18], nuclear factor erythroid 2-related factor 2 Nrf2 [19] or activator protein (AP)-1 [20]).

Although these studies have increased our understanding of emphysema development and progression, they have several potential limitations with respect to identification of precise molecular mechanisms. For example, the approaches were mostly focused on single gene analysis or characterization of cellular, inflammatory or histopathological changes without attempting a more comprehensive mechanistic investigation using multi-omics technologies. Remarkably, only a few studies have investigated global gene expression profiles associated with the development or progression of murine CS-induced emphysema [19, 21–24]. Considering that COPD is a highly heterogeneous and polygenic disease, a more comprehensive and powerful approach that translates these large-scale data sets into pathophysiological changes is essential to enhance understanding of the molecular mechanisms that induce alveolar destruction and subsequent emphysema. For instance, we recently applied Reverse Engineering and Forward Simulation (REFS™) technology to identify key molecular drivers of emphysema in A/J mice by integrating the whole genome expression data and COPD-relevant endpoints [25].

In the current study, we comprehensively investigated the lung transcriptomes leveraging the RCR [26] methodology to evaluate molecular mechanisms underlying emphysematous changes. Among the 39 predictions that were common to the five strains/genotypes investigated, we identified mechanisms known to be associated with emphysema including NF-κB signaling, Toll-like receptor (TLR4) signaling, and increased protein abundance of inflammatory mediators such as tumor necrosis factor (TNF)-α, colony stimulating factor 2 (granulocyte–macrophage) (CSF2), as well as several interleukins. More importantly, we predicted a series of mechanisms for which a role in emphysema development is not yet well documented, e.g. transcriptional activity of spleen focus forming virus (SFFV) proviral integration oncogene (SFPI1, aka.PU.1), signal transducers and activators of transcription (STAT)-1, CCAAT/enhancer-binding protein (C/EBP), forkhead box M1 (FOXM1), Yin and Yang (YY)-1, nuclear receptor corepressor (N-COR), increased protein expression of IL-17, or decreased protein expression of integrin Beta-6 (ITGB6) and cystic fibrosis transmembrane conductance regulator (CFTR). The present study contributes to a better understanding of the specific CS-induced molecular processes underlying emphysema development in mice by elucidating novel effectors implicated in disease progression, which could also be relevant in human COPD.

## Materials and methods

### Transcriptomics data sets

Transcriptomics data sets were derived from four mouse CS-inhalation studies: C57BL/6 [27], apolipoprotein E–deficient (ApoE^−^/^−^) C57BL/6 mice [28], A/J mice [25], CD1 and nuclear factor erythroid 2-related factor 2-deficient CD1 mice (Nrf2^−^/^−^) [29]. The exposure studies, three of which have been published in detail previously, were conducted in Philip Morris Research Laboratories at different times and locations under similar mainstream CS exposure protocols (Table 1). RNA was isolated from either whole lung (C57BL/6, ApoE^−/−^, Nrf2^−/−^, and CD1 mice) or lung parenchyma by laser capture microdissection (A/J and ApoE^−/−^ mice). In case of C57BL/6 mice, genome-wide expression profiling of whole lung was conducted at 1, 3, 5, and 7 months in the sham (air exposed) and CS groups (for experimental details, see below). Affymetrix CEL files that passed the quality check were used for further data processing and are publicly available: Gene Expression Omnibus (GSE18344) for the CD1/Nrf2^−^/^−^ mice, Array Express (E-MTAB-1390) for the C57BL/6 ApoE^−^/^−^ mice, Array Express (E-MTAB-1426) for the A/J mice, and Array Express (E-MTAB-2756) for the C57BL/6 mice.

**Table 1:** Comparison of four cigarette smoke exposure studies

Mouse strain Provider	C57BL/6 Charles River, USA	ApoE^−/−^ (C57BL/6 background) Taconic, Denmark	A/J The Jackson Laboratory, USA	Nrf2^−/−^ (CD1 background) and CD1 RIKEN BRC, Japan
Age at start (weeks)	~8–9	~5–9	~10–15	~12–13
Treatment	Fresh air/3R4F	Fresh air/3R4F	Fresh air/3R4F	Fresh air/2R4F
Exposure duration				
Hours/day	4	3	4	4
Days/week	5	5	5	5
Months	1, 3, 5, 7	6	5	5
Smoking parameters	Health Canada Intense (HCI)	Health Canada Intense (HCI)	ISO 3308	ISO 3308
Concentration (mg TPM/m^3^)	750	600	750	750
Origin of gene expression data	Whole lung	Whole lung or lung parenchyma	Lung parenchyma	Whole lung
References	Hoeng et al. [27] and unpublished	Boue et al. [28]	Xiang et al. [25] and unpublished	Gebel et al. [29]
Dataset accession number and Database	E-MTAB-2756 ArrayExpress	E-MTAB-1390 ArrayExpress	E-MTAB-1426 ArrayExpress	GSE18344 GEO

*ApoE* apolipoprotein E, *Nrf2* nuclear factor, erythroid-derived 2, like 2

### Animals and CS exposure

Care and use of the mice was in conformity with the American Association for Laboratory Animal Science Policy on the Humane Care and Use of Laboratory Animals (http://www.aalas.org/). Animal experiments were approved by the Institutional Animal Care and Use Committee of Philip Morris Research Laboratories, Belgium or Singapore (C57BL/6 study).

Although the studies were conducted at different times, the smoke exposure conditions utilized in the studies were largely conserved (Table 1). The Standard Reference Cigarette 3R4F was obtained from the Tobacco and Health Institute at the University of Kentucky. It is a filter cigarette with reported mainstream smoke yields per cigarette of 11.0 mg total particulate matter (TPM), 0.73 mg nicotine, and 12.0 mg carbon monoxide (CO) (http://www2.ca.uky.edu/refcig/3R4F%20Preliminary%20Analysis.pdf). All cigarettes were conditioned and smoked according to International Organization for Standardization or Health Canada Intense parameters [28, 30, 31]. Mainstream CS was diluted with filtered conditioned air to a target concentration of 750 mg TPM/m^3^, unless otherwise noted (Table 1).

The C57BL/6 study was conducted as a seven-month smoke inhalation study using female C57BL/6 mice approximately 8–9 weeks of age at study commencement. Mice (eight per group) were exposed in whole-body chambers to diluted mainstream CS at a target concentration of 750 mg TPM/m^3^ for 4 h per day, 5 days per week, with intermittent exposure to fresh filtered air for 30 min after the first hour of smoke exposure and for 60 min after the second and third exposure hours to avoid a build-up of excessive carboxyhemoglobin (COHb) concentrations. An initial 2-week concentration adaptation period was implemented. Sham control animals were exposed to conditioned fresh air. Dissection was performed at the end of the scheduled exposure periods (1, 3, 5, and 7 months), 18–24 h after the last exposure. Lungs were snap-frozen, and for each lung tissue sample, 22 slices (each 20 µm) were cut using a cryostat and RNA was extracted, processed, and analyzed as previously described [25, 32].

The A/J mouse study [25] was conducted as a 5-month CS inhalation study using female A/J mice approximately 10–15 weeks of age at study commencement. Mice were exposed to diluted mainstream CS at a target concentration of 750 mg TPM/m^3^ for 4 h/day. After 5 months, lung parenchyma and airway samples were collected using laser-capture microdissection as described [33] from CS-exposed mice and sham control mice exposed to conditioned fresh air. The presence of emphysema was histologically confirmed.

The ApoE^−^/^−^ mouse study [28] was conducted as a six-month CS inhalation study using female ApoE^−^/^−^ mice approximately 5–9 weeks of age at study commencement. Animals were exposed to diluted mainstream CS at a target concentration of 600 mg TPM/m^3^. Following 6 months of exposure, whole lung and isolated parenchyma were collected from CS-exposed mice and sham control mice exposed to conditioned fresh air. The presence of emphysema was histologically confirmed.

The Nrf2^−^/^−^ study [34] was conducted as a 5-month smoke exposure study using both female Nrf2^−^/^−^ mice and wild-type littermates on the CD1 background that were approximately 12–13 weeks of age at study commencement. Animals were exposed to diluted mainstream CS at a target concentration of 750 mg TPM/m^3^ for 4 h/day, 5 days per week. Whole lung samples were collected after a 5-month CS-exposure. The presence of emphysema was histologically confirmed.

### Reverse causal reasoning

RCR interrogates a literature-based Knowledge Assembly Model (KAM; a collection of biological concepts and entities, and their causal relationships), to identify upstream controllers of gene expression changes (so called State Changes) observed in the transcriptomic data set [26]. For all of the data set analysis, the full Selventa Knowledgebase consisting of causal connections curated from multiple species and contexts (e.g. human, mouse, and rat) served as the KAM to interrogate the State Changes. The potential upstream controllers identified by RCR are termed “hypotheses” (HYPs) and are statistically significant potential explanations for observed mRNA State Changes. Each HYP is scored according to two probabilistic metrics, richness and concordance. Richness is the probability that the number of observed mRNA State Changes connected to a given HYP could have occurred by chance alone, calculated using the hypergeometric distribution. Concordance is the probability that the number of observed mRNA State Changes connected to the HYP occur in the proper direction as predicted in the KAM (e.g. increased or decreased activity, or abundance of a node), when compared to chance alone, calculated using the binomial distribution. The HYPs with *P* < 0.05 for richness and concordance were considered statistically significant.

To highlight patterns of significance of the concordance values over time or across mouse strains, common HYPs were grouped using a hierarchical clustering approach. The clustering was performed using the Euclidean distance as dissimilarity metrics calculated with the minus log_10_-transformed concordance *P* values between each pair of HYPs and the “median” as agglomerative method. The minus log_10_-transformed concordance *P* values were multiplied by the sign of the concordance scores to provide the directionality of regulation of the predicted upstream controller (HYPs).

### Lung histomorphometry

Alveolar emphysema was assessed by mean chord length (Lm) measurements, as described previously [28]. Briefly, several step serial sections per animal, representing a cross section along the left main stem bronchus and its branching bronchioles, was selected for morphometric evaluation. Lm measurements were performed on images captured with an Axio-Imager-Z1 microscope equipped with an 8-specimen holder, a high-resolution digital color camera (Olympus DP70) and VIS-software from Visiopharm (Horsholm, Denmark). All measurements were conducted according to Visiopharm’s quantitative digital pathology methods.

### Bronchoalveolar lavage fluid (BALF) collection and analysis

BALF was collected as previously described [28]. Cell-free BALF supernatants were analyzed by Myriad Rules-Based Medicine (Myriad RBM, Austin, TX, USA) using a Rodent MAP 2.0 antibody array with 59 analytes/biomarkers (http://rbm.myriad.com/products-services/rodentmap-services/rodentmap/). Analyte measurements below the least detectable dose (LDD) or the lower limit of quantification (LLOQ) were substituted by max (LDD, LLOQ) and divided by two. Six analytes (FGF-9, GST-alpha, IFN-gamma, IL-17A, IL-3, and SGOT) were excluded from the analysis since they were largely below the detection/quantification limits.

### Reverse phase protein array (RPPA) analysis

The TissueLyser II (Qiagen, Hilden, Germany) bead-mill disruption system was used for protein extraction from cryo-sliced right lung tissue samples, which were obtained from a confirmatory follow-up study with the same exposure conditions. Protein extraction was performed according to the manufacturer’s instructions, using one 5-mm steel bead (Qiagen) and 400 µl Zeptosens cell lysis buffer (CLB1, Bayer Technology Services GmbH, Leverkusen, Germany). After extraction, the sample was centrifuged in a micro-centrifuge for 10 min at 14,000 rpm, and the cleared supernatant was transferred into fresh tubes for further analysis. Protein concentration of collected supernatants was quantified as described [35] using a fluorescence-based protein microspot assay [EZQ™ Protein Quantitation Kit (Life Technologies™, Logan, UT, USA)]. Protein extracts were stored at −80 °C until further use.

Analysis of protein extracts by Zeptosens^®^RPPA technology was performed as previously described [36]. Protein extracts were adjusted to uniform concentrations with CLB1 buffer. Adjusted extracts were further diluted with CLB1 buffer to a final spotting concentration of 0.1 µg/µl. Diluted extracts were printed at four serial dilutions (1.6-fold) onto Zeptosens^®^ hydrophobic chips (Bayer Technology Services GmbH) using a microarray printer (NanoPlotter 2.1, GeSiM, Grosserkmannsdorf, Germany). Each sample was processed in technical duplicates with independent dilutions on separate arrays, i.e. eight spots per sample with four spots per array. Following array printing, arrays were blocked, washed, and dried according to the manufacturer’s specifications and stored at 4 °C in dark until further use.

For measurement of protein signals, arrays were incubated overnight at room temperature in primary antibodies at dilutions of 1:500 in CAB1 assay buffer (Bayer Technology Services GmbH). The following primary antibodies were used: anti-BRCA1 (sc646, Santa Cruz Biotechnology, Dallas, TX, USA), anti-C/EBPα (sc61, Santa Cruz Biotechnology), anti-C/EBPβ (sc150, Santa Cruz Biotechnology), anti-ITGB6 (sc15329, Santa Cruz Biotechnology), anti-PU.1 (LSC30679, LifeSpan BioSciences, Seattle, WA, USA), anti-SOCS3 (04004, Merck Millipore, Darmstadt, Germany), anti-STAT1 (9172, Cell Signaling Technologies, Danvers, MA, USA), and anti-STAT1(Tyr701) (44376G, Life Technologies). Following washing, arrays were incubated for 2 h with either Alexa647- (Life Technologies) or Dylight650- (Bio-Rad AbD Serotec GmbH, Puchheim, Germany) labeled anti-species secondary antibodies, at dilutions of 1:500. After subsequent washing, stained arrays were imaged using an array scanner (ZeptoREADER, Bayer Technology Services GmbH), according to the manufacturer’s recommendations. For correction of anti-species secondary antibody staining, arrays were assayed in the absence of primary antibodies. For measurement of spotted protein amounts, one blank chip (i.e. without antibody incubation) was stained with SYPRO^®^Ruby. Scanned images were analyzed using ZeptoVIEW 3.1 software (Bayer Technology Services GmbH). Normalized fluorescence intensities (NFI) for each sample and protein target were calculated as reference fluorescence intensities of primary antibody stained arrays (RFIprimary) corrected for secondary antibody staining (RFIsecondary), as well as relative spotted protein concentration (RFIprotein), determined by (RFIprimary–RFIsecondary)/RFIprotein, using the Zepto VIEW 3.1 software. NFI values were used for subsequent analysis.

### Statistical analysis

For BALF analysis (MAP), RPPA, and histomorphometry, two-tailed *t* tests were performed, assuming unequal variance.

## Results

### Identification of potential mechanistic explanations of CS-induced emphysema

**Fig. 1:**
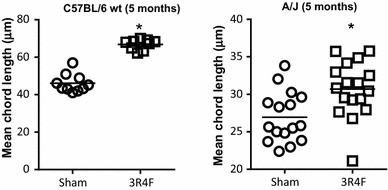
Mean chord length (Lm) in C57BL/6 (*left*) and A/J (*right*) mice exposed to fresh air (sham) or cigarette smoke (3R4F) for 5 months. Individual values are presented (*n* = 10–17 per group). The exposure regime is described in “Materials and methods”. *P ≤ 0.05 (3R4F vs. sham)

To determine the molecular mechanisms underlying CS-induced lung emphysema, we applied RCR to transcriptomic data sets derived from lungs of five mouse genotypes (C57BL/6, ApoE^**−/−**^, A/J, Nrf2^**−/−**^ and CD1), susceptible to emphysema development following a prolonged (5–6 months) exposure to CS. We and others have already demonstrated that exposure to CS leads to increased mean chord length (Lm) in CD1 (12.5 %), Nrf2^−/−^ (16 %), and ApoE^−/−^ (17.1 %) mice [19, 28, 29]. In addition, long-term exposure to CS (5 months) resulted in significant increase in the Lm in A/J (14 %) and C57BL/6 (44.9 %) mice (Fig. 1).

**Fig. 2:**
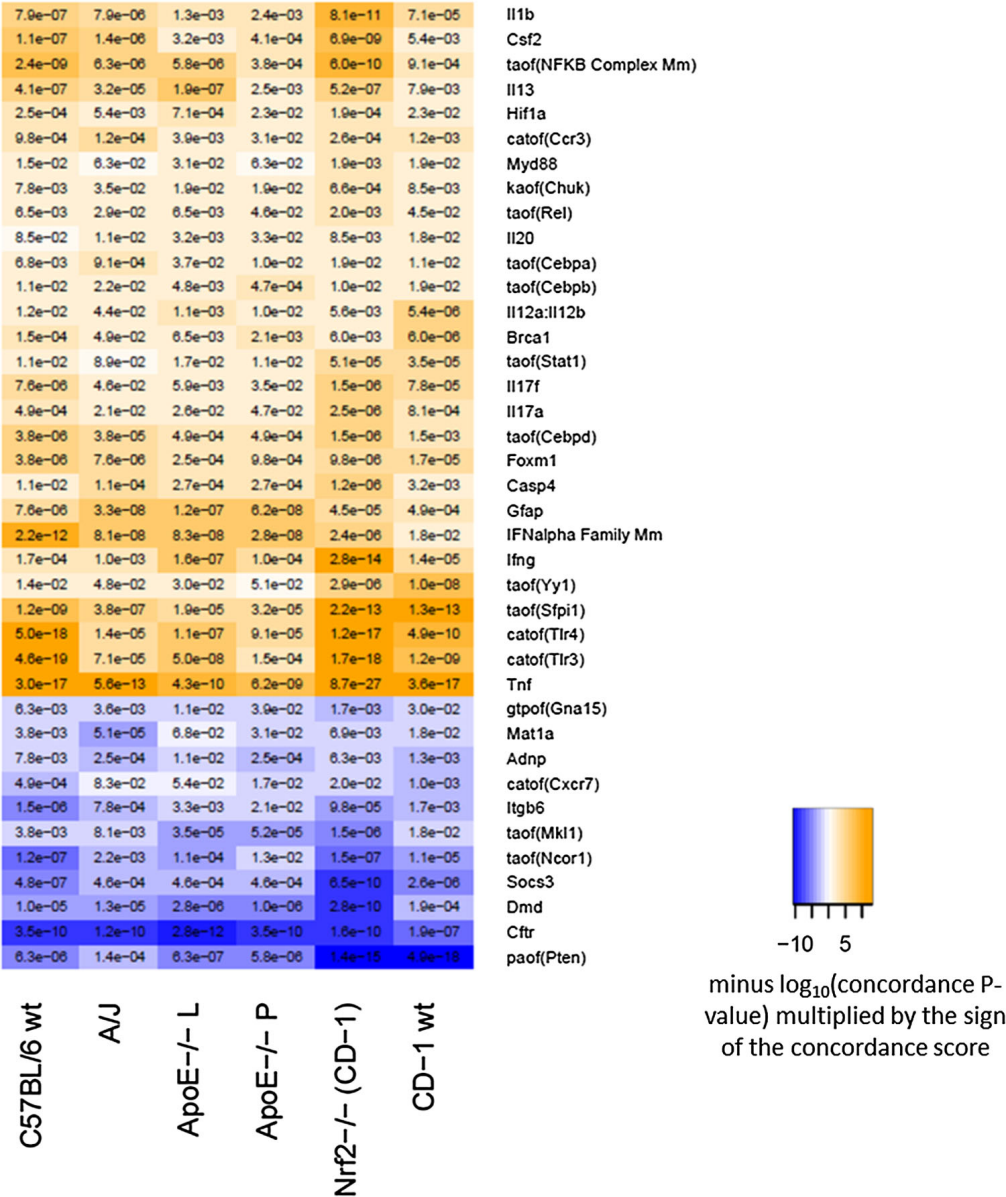
Common HYPs across five mouse models exposed to cigarette smoke for 5–6 months. Results are shown as hierarchically clustered color-coded heatmap according to HYP concordance and richness (3R4F/2R4F vs. sham comparison is shown). *Yellow-orange* to *blue* gradient indicates predicted increase and decrease in abundance or activity of HYPs. Catof, catalytic activity of; kaof, kinase activity of; taof, transcriptional activity of; paof, phosphatase activity of; gtpof, GTP-binding activity of. L—whole lung, P—lung parenchyma (prepared by laser capture microdissection) (color figure online)

As described previously, RCR is a computational methodology that uses a set of differentially measured biological entities (e.g. mRNA) as inputs to make predictions (called “HYPs”) about the identity of potential upstream controllers of observed differential measurements [26]. For example, HYPs can refer to biological entities such as protein abundance, regulatory mechanisms such as activities of receptors, transcription factors, or kinases. By comparing six data sets, we identified 39 predicted HYPs that were conserved across five genotypes (Fig. 2, Supplementary File 1). Another 37 common HYPs (Supplementary File 1) were also predicted to be regulated. They were related to biological processes (e.g. Natural killer cell activation, T-helper 2 cell differentiation) or were proxies that represented mechanisms using experimental perturbations (Ovalbumin, Sirolimus, Bleomycin, etc.), and were not currently considered for the experimental verification. Out of the selected HYPs related to biological entities, 28 were predicted to increase, and 11 predicted to decrease. The majority of HYPs were related to the inflammatory response and mapped to nine sub-networks, lung-specific Inflammatory Process Network model [37] and Tissue Repair and Angiogenesis Network model [38], consisting of the following submodels: Tissue Damage, Neutrophil Response and Chemotaxis, Mucus Hypersecretion, Macrophage Mediated Recruitment, Macrophage Activation, Immune Regulation of Tissue Repair, Immune Regulation of Angiogenesis, Epithelial Proinflammatory Signaling, and Dendritic Cell Activation (Table 2). More specifically, among the common HYPs, we identified increased protein abundance of inflammatory mediators associated with T helper (Th) responses [e.g.TNF-α, IFN-γ, IL-1β, colony stimulating factor (CSF)-2, IL-13, IL-17A, and IL-17F]; increased activity of inflammation-related TFs [e.g. NF-κB, STAT-1, Sfpi1 (known as PU.1)], C/EBP family members α, β, and δ; and increased receptor activity of CCR3 and Toll-like receptors (TLRs)3 and 4.

**Table 2:**
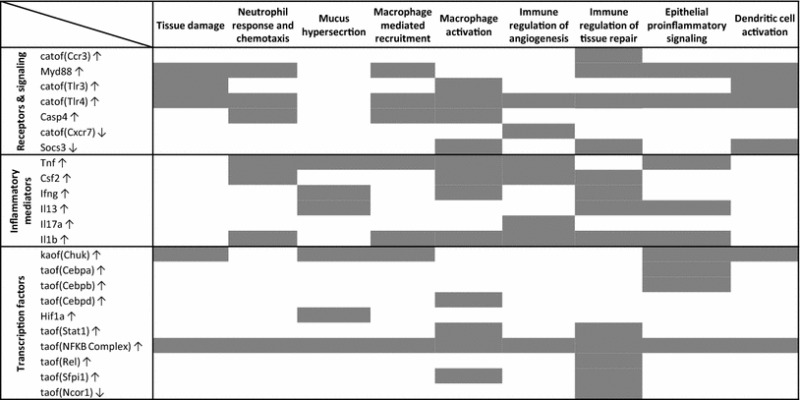
Common HYPs mapped to subnetworks of the Inflammatory Process Network. Presence of a HYP within a specific subnetwork is indicated by a gray block. Catof, catalytic activity of; kaof, kinase activity of; taof, transcriptional activity of. ↑, increase; ↓, decrease

### Time-course of the occurrence of common mechanisms in C57BL/6 mice

**Fig. 3:**
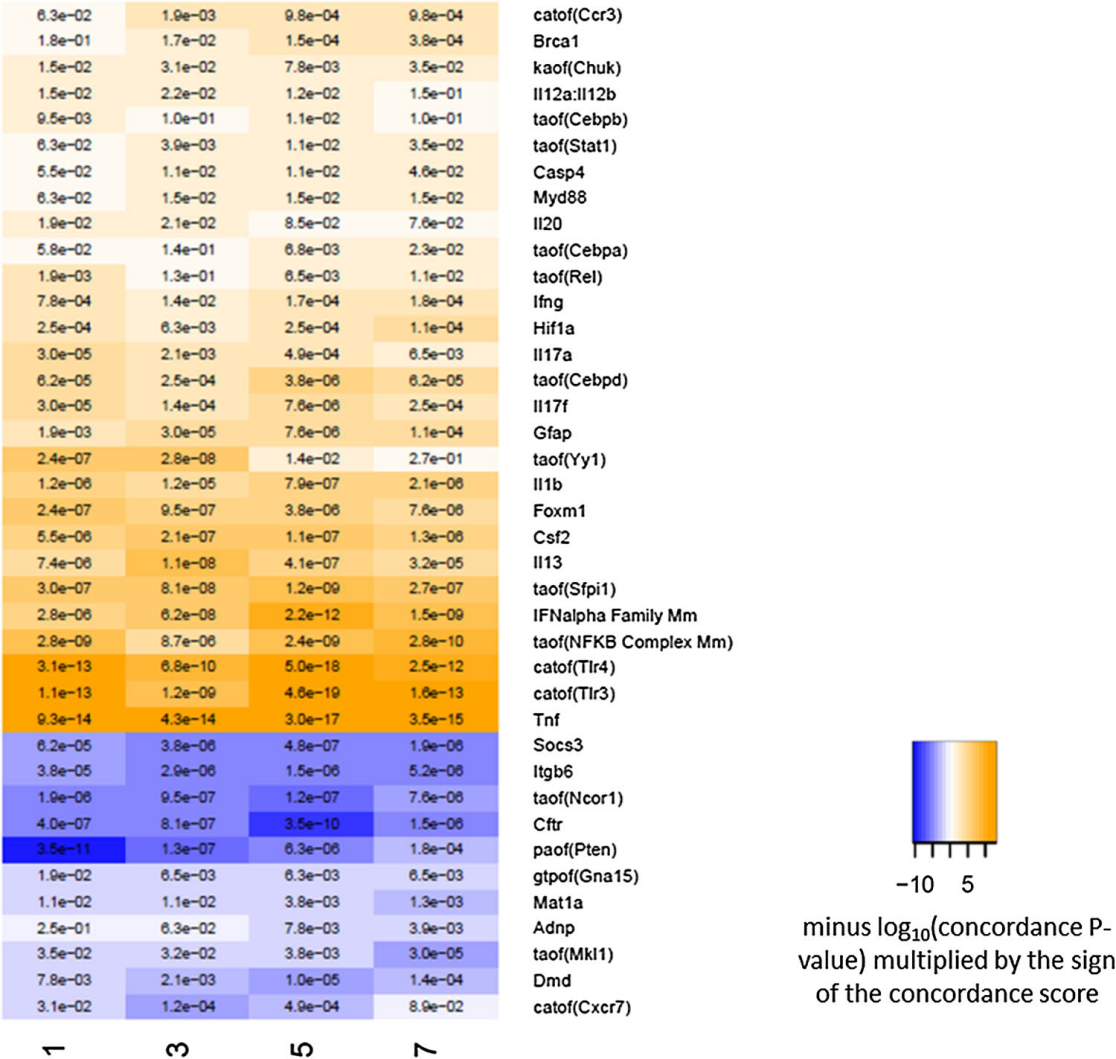
Time course of the occurrence of common HYPs in C57BL/6 mice exposed to cigarette smoke (3R4F) for up to 7 months. Results are shown as hierarchically clustered color-coded heatmap according to HYP concordance and richness (3R4F vs. sham comparison is shown). *Yellow-orange* to *blue* gradient indicates predicted increase and decrease in abundance or activity of HYPs. Catof, catalytic activity of; kaof, kinase activity of; taof, transcriptional activity of; paof, phosphatase activity of; gtpof, GTP-binding activity of. Note: For an easier tracking of the common HYPs from the strain comparison (Fig. 2) and from the temporal development in one strain (Fig. 3), a side-by-side comparison in alphabetical order of all HYPs from both heatmaps has been provided in Supplementary Figure 1 (color figure online)

Since the comparative RCR analysis was performed cross-sectionally on data sets derived from lungs with fully developed emphysema, i.e. at the five-month time-point, we investigated longitudinally the time-course of these commonly identified HYPs as disease-related mechanisms in one strain, i.e. the C57BL/6, from months one to seven. To elucidate whether the predicted mechanisms might be associated with the onset of destructive lung lesions, we analyzed transcriptomic data sets obtained from lungs of air- or smoke-exposed C57BL/6 mice at early time points (months 1 and 3) preceding and accompanying the occurrence of observable anatomic changes associated with emphysema ([21, 39] and unpublished data). Nearly all the HYPs identified in the emphysematous lungs (at 5–6 months) were already statistically significant after the first month of CS exposure (Fig. 3, Supplementary Figure 1), suggesting they could be linked to emphysema onset and progression. Moreover, the majority of HYPs (including increased catalytic activity of TLRs, transcriptional activity of NF-κB, PU.1 and C/EBP, and protein abundance of IFN-γ, CSF2, IL-1β, and TNF-α) remained significantly predicted over the course of CS exposure. Some HYPs, however, such as receptor activity of CCR3 and CCR7, transcriptional activity of C/EBPα and STAT1, and increased protein expression of BRCA1, CASP4, and MyD88 were statistically significant from month 3 onwards of CS exposure. Other HYPs were significant at early time points and not at later time points, namely transcriptional activity of YY1 and protein abundance of IL-20. Phosphatase activity of phosphatase and tensin homolog (PTEN) remained significant with up to 7 months of CS exposure, when compared with fresh air-exposed mice. In the case of suppressor of cytokine signaling (SOCS)-3, ITGB6, and CFTR, predicted decrease in their protein expression persisted over time.

### Proteomic analysis of selected HYPs in C57BL/6 mice

**Fig. 4:**
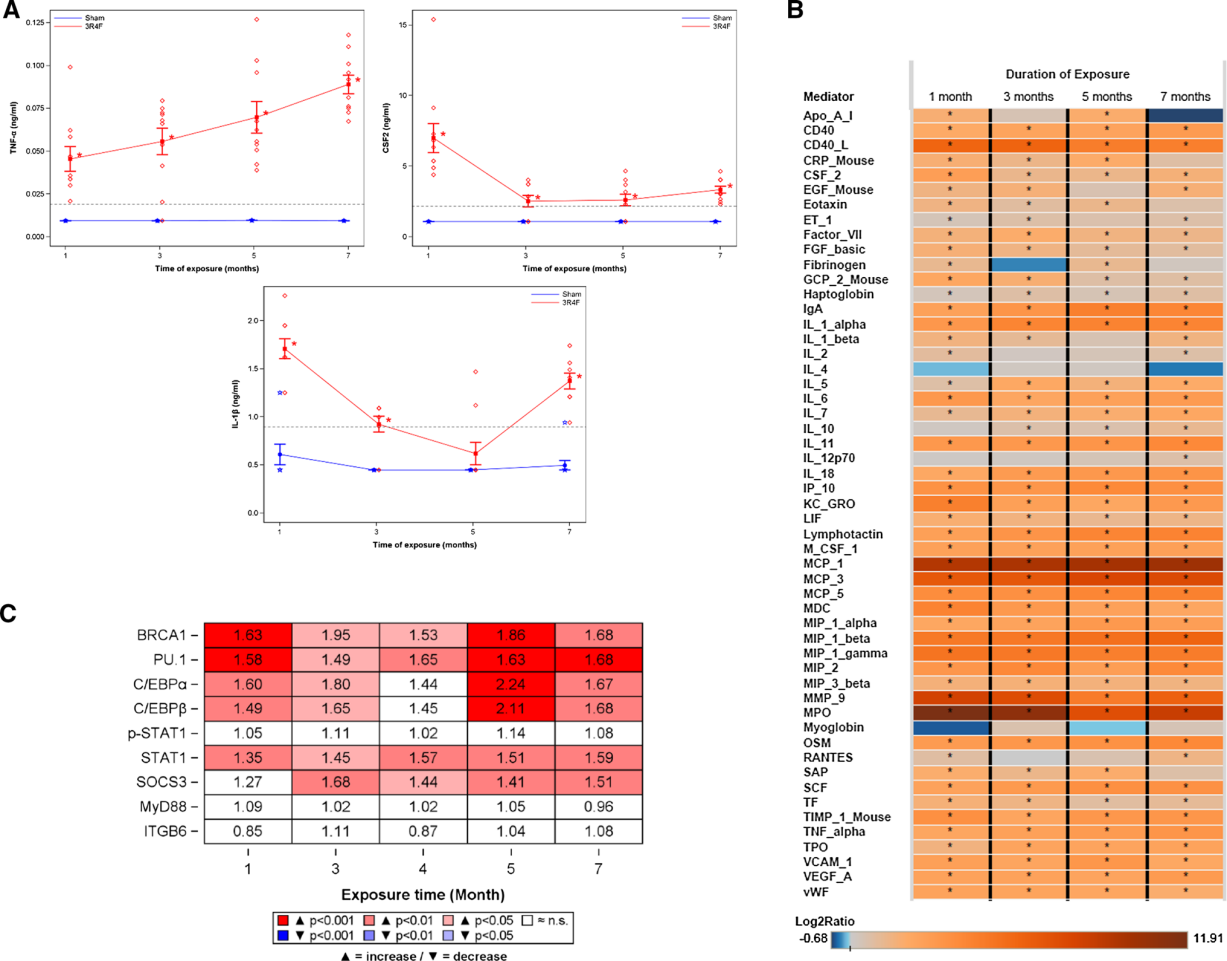
Proteomic analysis of selected HYPs in C57BL/6 mice exposed to fresh air (sham) or cigarette smoke (3R4F) for up to 7 months. C57BL/6 mice were exposed as described in “Materials and methods” and killed 18–24 h after the last exposure. (**a**) Levels of BALF inflammatory mediators were measured using multiplexed bead array as described in “Materials and methods”. Values of selected inflammatory mediators are presented as single measurements and as mean ± SD (*n* = 8–10 per group). *P ≤ 0.05; (3R4F vs. sham). *Blue stars* and *lines* sham; *red diamonds* and *lines* 3R4F; *gray dotted line* limit of detection/quantification (**b**) Heatmap representation of all regulated inflammatory mediators from the BALF analysis. The* color coding* indicates the Log_2_ ratio of protein abundance (3R4F vs. sham); *P ≤ 0.05. (**c**) Protein abundance in lung homogenates was measured using RPPA as described in “Materials and methods” (*n* = 10 per group). Results are shown as ratios of abundance (3R4F/Sham) and color-coded heatmap reflecting statistical significance (color figure online)

Next, we applied two targeted proteomic approaches to further explore our findings using lungs of C57BL/6 mice exposed to fresh air or 3R4F for up to 7 months. BALF was analyzed with multiplexed bead arrays (Fig. 4a, b), whereas RPPA was applied to lung tissue homogenates from a follow-up study with the same design (Fig. 4c). For RCR predictions reflecting inflammatory mediator protein abundance, we confirmed that BALF levels of TNF-α, Il-1β, CSF2 (Fig. 4a), and other “non-HYP” inflammatory mediators including several interleukins, myeloperoxidase (MPO), and matrix-metalloproteinase-9 (MMP-9) (Fig. 4b) were increased upon CS exposure. Protein abundances particularly related to six transcriptional activity-type HYPs were analyzed in lung homogenates by measuring increased protein abundance and phosphorylation levels (Fig. 4c). We applied this approach based on the assumption that regulatory activity (such as DNA-binding ability) of a TF strongly depends on its protein abundance [40, 41]. We observed increased protein levels of BRCA1, C/EBPα, C/EBPβ, PU.1, and STAT1, thereby confirming the computationally predicted protein candidates; however, we did not detect increased tyrosine (Tyr705) phosphorylation levels of STAT1 that would further indicate its transcriptional activation. In contrast to RCR predictions, SOCS3 protein expression was increased, whereas MyD88 and ITGB6 protein abundances were not affected by CS exposure (Fig. 4c).

## Discussion

**Fig. 5:**
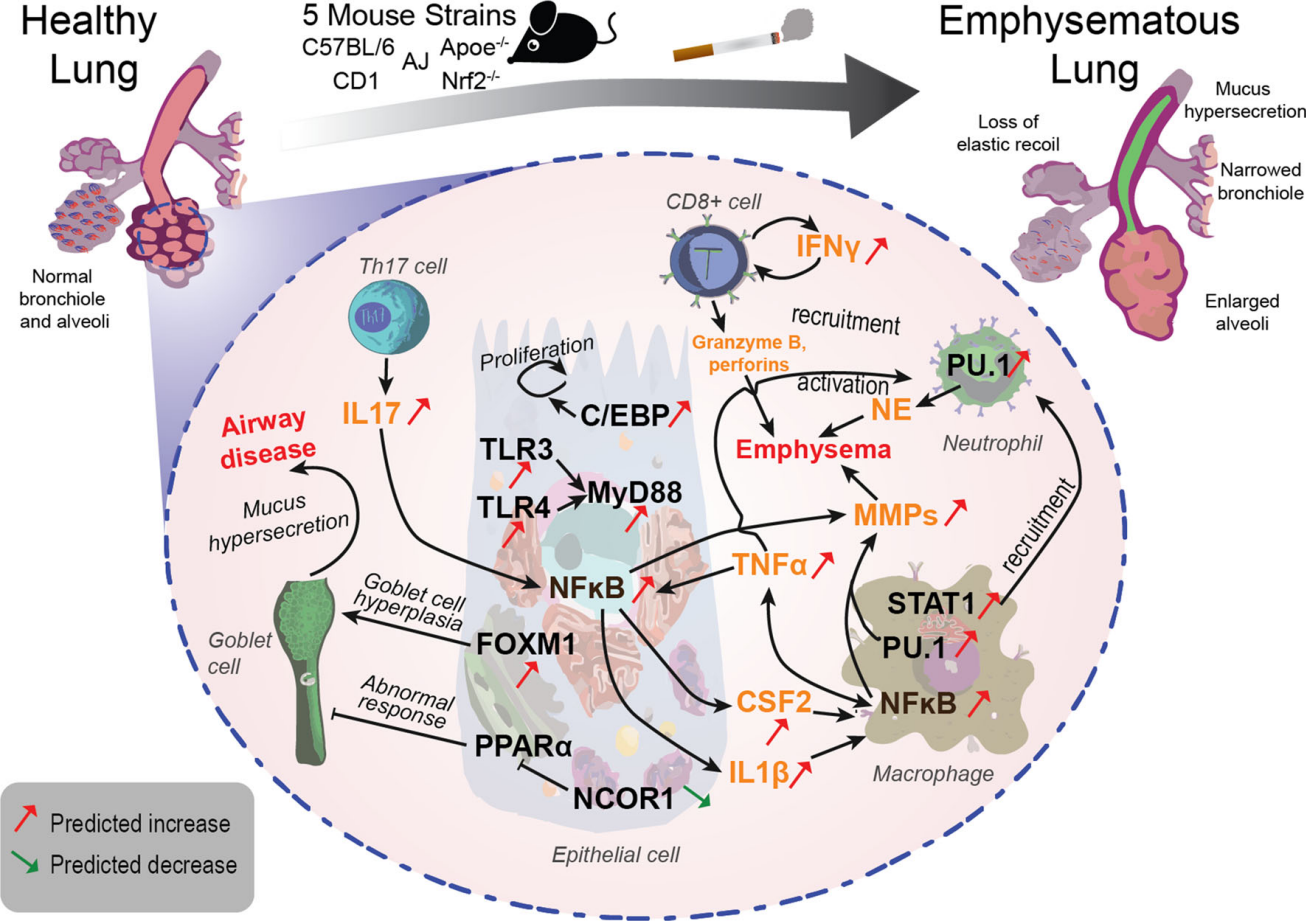
Possible interrelationships and roles for the identified common mechanisms (HYPs) in five mouse models of emphysema in a framework of classical human COPD mechanisms. The RCR-predicted decreases (*green arrows*) and increases (*red arrows*) in transcription factors (*black font*) and inflammatory mediators (*orange font*) common to the investigated mouse models have been associated with classical pathways of human COPD pathogenesis (*black arrows*) as depicted, e.g., in recent reviews [98, 99] and in other relevant articles mentioned in the “Discussion”. Central predicted roles for emphysema formation (on the right side) are assigned to the NFκB signaling in epithelial cells and macrophages triggered by, e.g., TNFα, IL17, or activation of TLR3/4, resulting in the shedding of TNFα, CSF2, Il1b, and MMPs. The predicted and measured activation of STAT1‐ and PU.1 would (particularly in macrophages) also be consistent with the recruitment and activation of neutrophils that enhance emphysema by NE production. On the left side of the scheme, the predicted decrease in NCOR1 activity in conjunction with increased transcriptional activity of FOXM1 would be in conformity with goblet cell activation involved in airway disease (which in mouse models is less pronounced than in human COPD patients). For further details, please refer to the “Discussion” (color figure online)

In this study, we employed RCR on whole-genome transcriptomic data sets to computationally predict molecular mechanisms associated with the development of an emphysematous phenotype in mouse models of disease. We predicted 39 HYPs that may represent molecular mechanisms associated with the development of CS-induced emphysema. These HYPs were conserved across five susceptible mouse models (C57BL/6, ApoE^**−/−**^, A/J, CD1, and Nrf2^**−/−**^), suggesting that a core set of biological features may be closely related to emphysema development in a mouse model and may potentially be translatable to human disease. Encouragingly, we verified various well-known mechanistic changes associated with emphysema development, such as NF-κB signaling and TLR4 signaling, as well as increased levels of TNF-α, CSF2, and non-predicted but related interleukins, MPO, and MMP-9 by means of proteomic analysis. Even more importantly, we identified several novel molecular mechanisms, such as increased transcriptional activity of PU.1, STAT1, C/EBP, FOXM1, and YY1, decreased activity of N-COR1, and reduced protein abundance of ITGB6 and CFTR which have been implicated in disease progression but have not been comprehensively studied in this context, and can be investigated further by targeted experiments. Of note, a large proportion of the HYPs were related to inflammatory processes as outlined in Fig. 5, consistent with the current general concept that an abnormal inflammatory response is a crucial pathological mechanism in the development of CS-induced emphysema [42–44].

### Transcription factors

Transcriptional regulators of inflammatory genes including those related to cytokines, adhesion molecules, enzymes and receptors, is part of the molecular signaling that drives the development and progression of lung pathologies such as asthma and COPD [45, 46]. Therefore, not surprisingly, we predicted increased activation of TFs with well-known roles in emphysema development, such as NF-κB [10, 45, 47–52], but we also revealed TFs with less described or unknown roles in the etiology of emphysema.

For instance, we predicted increased transcriptional activity of C/EBP family members (α, β, and δ) and detected increased abundance (which may be consistent with an effective increase in transcriptional activity) of C/EBPα and β proteins following smoke exposure. The C/EBP family members are key transcriptional regulators of cell differentiation, proliferation, apoptosis, and inflammatory responses [53–55]. Although the binding activity of C/EBPβ, and to a lesser extent of C/EBPα, was enhanced in smokers compared with never-smokers, their role in emphysema remains undefined. It has been hypothesized that CS-increased C/EBP activity serves as a protective mechanism by stimulating lung epithelial regeneration through promotion of cell proliferation, inhibition of apoptotic signals, and activation of inflammatory genes [53]. In mice, lung epithelial-specific inactivation of C/EBPβ resulted in impaired CS-induced neutrophil recruitment to the lung and compromised induction of neutrophil chemoattractants, but its contribution to emphysema is unclear, in particular because the mRNA levels of C/EBPβ were down-regulated in the airway epithelium of smokers [56], which is difficult to align with the previous findings from the same group that the C/EBP binding activities were increased in the lung tissue from healthy smokers but not from COPD and chronic bronchitis patients [53]. Our results, together with the known role of C/EBPs in controlling expression of genes encoding inflammatory cytokines, and proteinases, suggest C/EBP family members as important players in the pathogenesis of emphysema [53, 54, 57].

We also predicted increased activity of PU.1, a TF selectively expressed in hematopoietic tissues, which plays a critical role in development, maturation, and function of lymphoid and myeloid cells such as macrophages [58]. Indeed, PU.1 is required for many functions performed by alveolar macrophages, including phagocytosis, MMP expression, reactive oxygen species production, surfactant catabolism, and secretion of pro-inflammatory cytokines [58] and lipoxygenase, which is implicated in asthma, atherosclerosis, and arthritis [59]. In addition, PU.1 has been shown to regulate expression and activity of emphysema-associated proteolytic enzymes such as cathepsin S and neutrophil elastase (NE) [60, 61]. Our results suggest that the activity of PU.1 may play a significant role in the development and progression of emphysema due to its maximal statistical enrichment occurring at month 5 (Fig. 3), corresponding to the time course of increasing emphysematous changes in the C57BL/6 model [62]. Furthermore, its activity is even more pronounced in other mouse strains (e.g. Nrf2^−^/^−^) at the month-5 time point, suggesting an even more significant role in the process of emphysema development (Fig. 2).

STAT1 is a known inducer of emphysema-associated processes such as apoptosis and inflammation; however, its precise role in emphysema and COPD development has not been established. Recently it has been demonstrated that STAT1 protein abundance is augmented in emphysematous lungs of gp130^F/F^ mice, although without significant changes in its tyrosine phosphorylation [63]. In addition, exposure of bronchial cells to CS extracts resulted in increased STAT1 phosphorylation and binding to the promoter regions of intercellular adhesion molecule (ICAM)-1, a mechanism that may contribute to airway neutrophilia and tissue damage [64]. STAT1 phosphorylation levels are increased in sputum from COPD patients, which induces oxidative and nitrosative stress in 16-HBE bronchial epithelial cells [65]. In bronchial biopsies from COPD patients compared to asymptomatic smokers, more cells being positive for phosphorylated STAT1 have been observed [66]. Results described here further support a potential role of STAT1 in emphysema development.

We also predicted increased transcriptional activity of FOXM1 which is a member of the forkhead box (Fox) family of TFs that play important roles in cellular proliferation, differentiation, and tumorigenesis [67]. Its role in inflammation has been evaluated using a mouse model of house dust mite allergen-induced allergic asthma. It has been shown that conditional deletion of the *Foxm1* gene from either airway epithelium or myeloid inflammatory cells decreased goblet cell metaplasia, reduced lung inflammation, and decreased airway resistance [68]. Moreover, the amount of FOXM1 protein was increased in bronchiolar epithelium and in inflammatory cells isolated from COPD patients [68], but no further studies were performed to investigate the role of FOXM1 in CS-induced emphysema in mice or humans. For YY1, we also predicted increased transcriptional activity predominantly at the early time points and this effect was diminished at later time points; so far, a role for this transcription factor in COPD has been observed only in COPD-associated skeletal muscle atrophy, but not in lung tissue [69]. No association with COPD has been reported for NCOR1, a co-repressor of PPAR δ, γ, and an antagonist of β-catenin [70], but the known involvement of the Wnt β-catenin pathway in abnormal airway responses in COPD patients [71, 72] and the established protective role of PPARγ activation against COPD [73–75] warrant further investigations related to the predicted activation of NCOR1 in COPD-susceptible mouse models.

### Th1/Th2/Th17-type cytokines

A common characteristic of COPD is progressive lymphocyte infiltration into the small airways and alveolar walls [76, 77]. In mice, CS-exposure induces lung recruitment of CD4^+^, CD8^+^, and B cells, enhancing pro-inflammatory Th1 cytokine production that might significantly contribute to emphysema development [8]. Indeed, deletion of genes encoding the TNF-α receptor or IFN-γ protects mice from development of CS-induced emphysema [78, 79], whereas TNF-α or IFN-γ overexpression leads to lung emphysema [80–82]. In this regard, using an RCR analysis and proteomic approach, we predicted and/or measured increased protein abundance over time for Th1-associated cytokines such as TNF-α, IL-2, and IFN-γ in CS-exposed mice. At the same time, we predicted increased protein levels of IL-13, a Th2 cytokine, which may contribute to mucus hypersecretion and chronic bronchitis in smokers and COPD patients [83, 84]. We also measured increased abundance of the Th2 effector cytokines IL-4, 5, 6, and 10. Although the mucus hypersecretion response is not well recapitulated in rodents [83], a direct role of IL-13 in CS-induced emphysema has been demonstrated [60].

A large number of studies have shown that COPD is largely associated with increased Th1 responses; however, recent studies have proposed an important role for Th17 cells in the pathogenesis of COPD [85, 86]. In mice, chronic exposure to CS has been shown to induce lung recruitment of Th17 cells [87]. More recently, Chen et al. [88] demonstrated that mice lacking the IL-17A receptor (*Il17Ra*^−*/*−^) failed to develop emphysema after 6 months of CS exposure. Likewise, IL-17 was essential for elastase-induced emphysema formation and lung inflammation in C57BL/6 mice [89]. In agreement with these data, we have predicted increased abundance of the two IL-17 family members, IL-17A and IL-17F, in emphysematous lungs, with maximal inferred abundance peaking at the month-5 experimental time point, corresponding to the time course of emphysematous changes and suggesting involvement in the tissue destruction process.

### Protease–antiprotease imbalance

Protease–antiprotease imbalance is regarded as a prevailing mechanism in the development of mouse and human emphysema [90]. Key events leading to lung extracellular matrix degradation during this process involve inflammatory cell recruitment into the lungs and the release of an uncontrolled amount of proteases. Indeed, increased lung concentrations of NE, MMP-1, MMP-9, and MMP-12 have been found in CS-exposed mice and emphysema patients [91], and thus excessive proteolytic activity must be tightly regulated. Morris et al. [92] described an interesting mechanism regulating MMP-12 activity, showing that *Itgb6*-null mice lacking the β-subunit of the αvβ6 integrin, an integrin abundantly expressed by bronchiolar and alveolar epithelial cells, developed spontaneous age-related MMP-12-dependent emphysema. In accordance, we predicted reduced ITGB6 protein abundance as a potential mechanism contributing to CS-induced emphysema; however, the measured ITGB6 protein abundance in lung homogenates did not change as a result of CS exposure. We can speculate that post translational modifications may stabilize the integrin molecules at the sites of cell adhesion and, therefore, a down-regulation of newly synthesized ITGB6 as predicted will not necessarily translate into reduced amounts of total ITGB6 in the tissue; however, further studies are necessary to explore the observed discrepancy.

Our proteomic approach did not confirm RCR predictions for decreased SOCS3 and increased MyD88 protein abundances; however, considering their known role in inflammatory processes these mechanisms are potentially active. SOCS3 is an important negative regulator of inflammatory reactions, especially in IL-6-driven pathological conditions [93]. Moreover, mice with hematopoietic cell-specific deletion of the *Socs3* gene developed neutrophilia and a variety of inflammatory pathologies [94]. Thus, the predicted decrease of SOCS3 protein may significantly contribute to the observed progression of CS-induced pathologies (e.g. neutrophilia, lung emphysema). We can speculate that RPPA-detected increased SOCS3 protein levels in the whole lung tissue may represent a compensatory mechanism to limit inflammation and/or SOCS3 protein degradation mechanisms can be inhibited by CS. With regard to MyD88, it serves as an essential adaptor protein in TLR and IL1R signaling pathway [95], and MyD88 deficiency leads to spontaneous emphysema [96]. On the other hand, CS-induced neutrophilia was ablated in MyD88-deficient mice [97].

Collectively, the RCR analysis of lung transcriptomic data provides a robust approach for comparing and identifying molecular changes involved in the development of CS-induced emphysema, which was also corroborated by the independently measured protein abundances of selected HYPs. Many of the common mechanistic features identified here are consistent with prevailing theories of disease development in murine models of CS-induced emphysema, as well as in the pathogenesis of human COPD, testifying to the powerful predictive ability of the RCR approach in assessing disease development and progression. The putative correspondence of predicted changes in transcription factor activities and inflammatory mediator abundances with classical mechanisms of COPD pathogenesis [98, 99] has been depicted in Fig. 5. Furthermore, we extended our analysis to identify a series of novel molecular mechanisms with potentially causative roles in emphysema development for further exploration. In conclusion, a systems toxicology approach, including histopathology, transcriptomics, proteomics, and computational hypothesis generation, has been applied to investigate the molecular mechanisms of CS-induced emphysema in a variety of susceptible mouse strains. Clearly, further studies to functionally validate our predictions are needed; however, the results of our meta-analysis open new vistas to obtain more detailed insights into the pathogenic mechanisms of emphysema, and of COPD in general.

## Electronic supplementary material

Supplementary material 1 (PPTX 265 kb) Supplementary Figure 1. Common HYPs across five mouse models exposed to cigarette smoke for 5–6 months (left panel) and across time points in C57BL/6 mice (right panel). Results from Figure 2 and Figure 3 are shown as alphabetically ordered color-coded heatmap according to HYP concordance and richness (3R4F/2R4F vs. sham comparison is shown). Yellow-orange to blue gradient indicates predicted increase and decrease in abundance or activity of HYPs. Catof, catalytic activity of; kaof, kinase activity of; taof, transcriptional activity of; paof, phosphatase activity of; gtpof, GTP-binding activity of. L-whole lung; P-lung parenchyma (prepared by laser capture microdissection).Supplementary File 1: List of all HYPs in the RCR analysis across five mouse models (6 data sets) exposed to cigarette smoke for 5–6 months. Rows 3–78 contain the 76 HYPs that were common to all 6 data sets, and the HYPs in rows 3 to 41 (highlighted) have been selected for further analysis (Figs. 2 and 3) because they relate to abundance of proteins or activity of receptors and transcription factors

Supplementary material 2 (XLSX 185 kb)
