# Supplementary material for: Transcriptional profiling and targeted proteomics reveals common molecular changes associated with cigarette smoke-induced lung emphysema development in five susceptible mouse strains
Source: Inflamm Res. 2015 May 12;64(7):471–86. doi: 10.1007/s00011-015-0820-2 (PMC4464601; doi:10.1007/s00011-015-0820-2)
Supplement: Supplementary file 1 — Supplementary material 1 (PPTX 265 kb) Supplementary Figure 1. Common HYPs across five mouse models exposed to cigarette smoke for 5–6 months (left panel) and across time points in C57BL/6 mice (right panel). Results from Figure 2 and Figure 3 are shown as alphabetically ordered color-coded heatmap according to HYP concordance and richness (3R4F/2R4F vs. sham comparison is shown). Yellow-orange to blue gradient indicates predicted increase and decrease in abundance or activity of HYPs. Catof, catalytic activity of; kaof, kinase activity of; taof, transcriptional activity of; paof, phosphatase activity of; gtpof, GTP-binding activity of. L-whole lung; P-lung parenchyma (prepared by laser capture microdissection).Supplementary File 1: List of all HYPs in the RCR analysis across five mouse models (6 data sets) exposed to cigarette smoke for 5–6 months. Rows 3–78 contain the 76 HYPs that were common to all 6 data sets, and the HYPs in rows 3 to 41 (highlighted) have been selected for further analysis (Figs. 2 and 3) because they relate to abundance of proteins or activity of receptors and transcription factors [file 11_2015_820_MOESM1_ESM.pptx]

## Slide 1
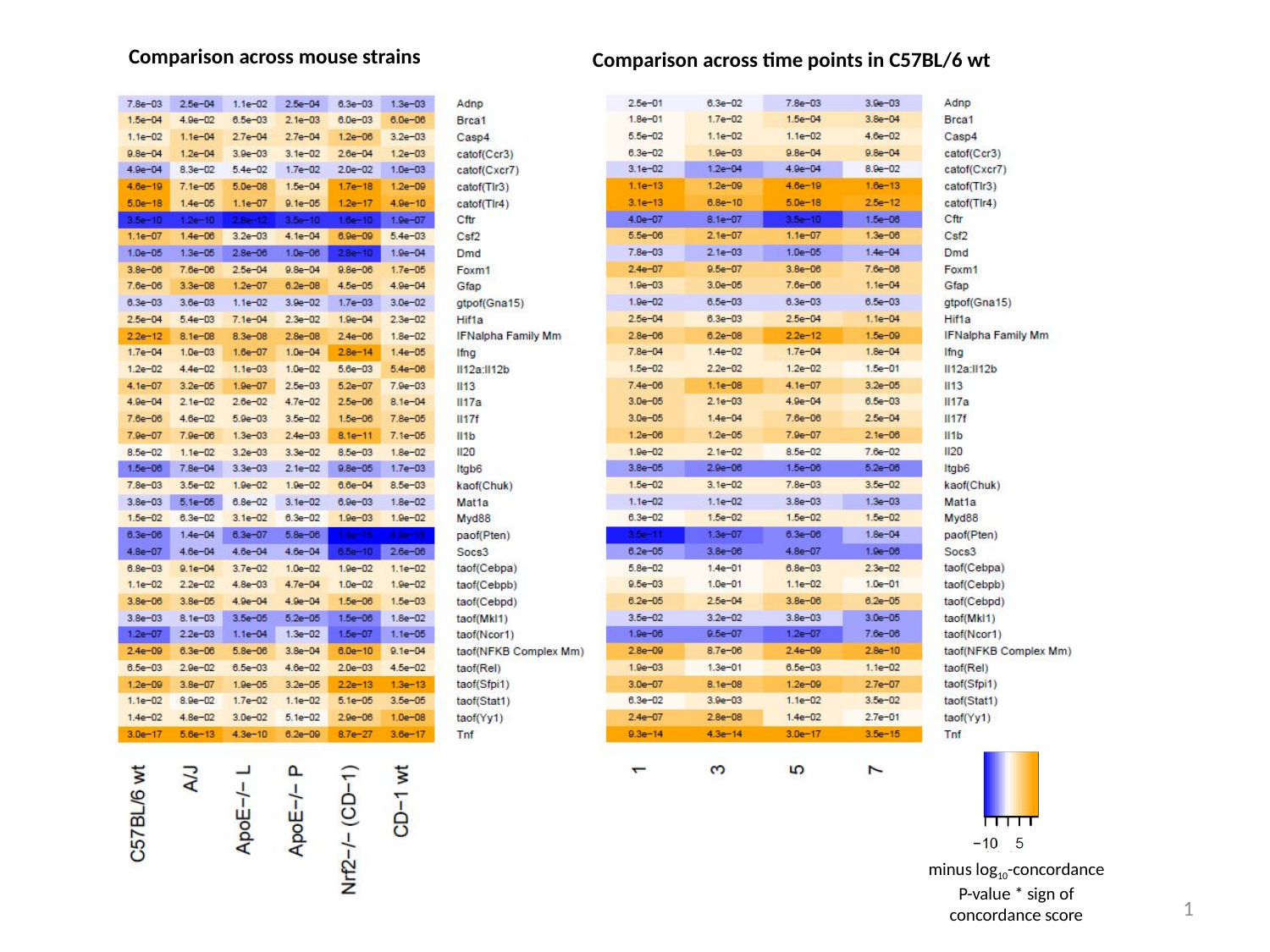

Comparison across mouse strains
Comparison across time points in C57BL/6 wt
minus log10-concordance P-value * sign of concordance score
1
